# Impact of Inhaled Corticosteroids on Growth in Children with Asthma: Systematic Review and Meta-Analysis

**DOI:** 10.1371/journal.pone.0133428

**Published:** 2015-07-20

**Authors:** Yoon Kong Loke, Patricia Blanco, Menaka Thavarajah, Andrew M. Wilson

**Affiliations:** Norwich Medical School, University of East Anglia, Norwich, United Kingdom; Beijing Institiute of Otolaryngology, CHINA

## Abstract

**Background:**

Long-term inhaled corticosteroids (ICS) may reduce growth velocity and final height of children with asthma. We aimed to evaluate the association between ICS use of >12 months and growth.

**Methods:**

We initially searched MEDLINE and EMBASE in July 2013, followed by a PubMed search updated to December 2014. We selected RCTs and controlled observational studies of ICS use in patients with asthma. We conducted random effects meta-analysis of mean differences in growth velocity (cm/year) or final height (cm) between groups. Heterogeneity was assessed using the I^2^ statistic.

**Results:**

We found 23 relevant studies (twenty RCTs and three observational studies) after screening 1882 hits. Meta-analysis of 16 RCTs showed that ICS use significantly reduced growth velocity at one year follow-up (mean difference -0.48 cm/year (95% CI -0.66 to -0.29)). There was evidence of a dose-response effect in three RCTs. Final adult height showed a mean reduction of -1.20 cm (95% CI -1.90 cm to -0.50 cm) with budesonide versus placebo in a high quality RCT. Meta-analysis of two lower quality observational studies revealed uncertainty in the association between ICS use and final adult height, pooled mean difference -0.85 cm (95% CI -3.35 to 1.65).

**Conclusion:**

Use of ICS for >12 months in children with asthma has a limited impact on annual growth velocity. In ICS users, there is a slight reduction of about a centimeter in final adult height, which when interpreted in the context of average adult height in England (175 cm for men and 161 cm for women), represents a 0.7% reduction compared to non-ICS users.

## Introduction

Inhaled corticosteroids (ICS) are amongst the most important treatment options in persistent asthma because of their efficacy in suppressing the inflammatory response. The clinical benefits of long-term therapy with ICS are consistently emphasized in national and international guidelines (United Kingdom and United States). [1, 2] However, there have been widespread, long-standing concerns regarding adverse events (such as fractures, and reduction of growth in children) with corticosteroids. [3, 4] The James Lind Alliance (in partnership with the British Thoracic Society and Asthma UK) reported on a recent prioritization exercise involving patients and health care professionals where adverse effects of corticosteroids was judged to be a top research priority. [5]

One of the major areas of concern and uncertainty is the potential for reduction in growth velocity and final height of children who are long-term users of inhaled corticosteroids (ICS). In 2006, Pedersen conducted a systematic review on randomized controlled trials (RCTs) of > 12 months ICS use in children, and concluded that there was possibly a small decrease in statural growth. [6] In an expert symposium, Skoner et al. noted a similar small decrease in growth, and an increase in cataracts, but argued that the earlier studies were methodologically weak, or based on drugs no longer in common use. [7]

Recent recommendations on comprehensive evaluations of adverse effects have suggested that a wide range of study designs (beyond just RCTs) may need to considered, depending on the features of the adverse outcome of interest. [8] Rare adverse events, or those that occur only after prolonged therapy, can be evaluated with non-randomized studies, perhaps even requiring a meta-analysis of various study designs. [8] Hence we aimed to analyse the effects of long-term (>12 months) ICS use in children with asthma, concentrating on growth velocity and final adult height in randomized and non-randomized studies.

## Methods

### Study Selection Criteria

As we were interested in long-term adverse effects that may be of relatively small magnitude, we selected studies with> 20 users of each ICS formulation, and follow-up of at least 52 weeks duration.

Our inclusion criteria for RCTs were (1) parallel-group RCT; (2) children with asthma of any severity; (3) ICS as the intervention vs a control treatment, where the comparison groups consisted of ICS vs other treatment, or ICS in combination with LABA vs a LABA alone; and (4) stated aim to evaluate growth velocity and/or final adult height.

We also evaluated controlled observational studies (case control, prospective cohort or retrospective cohort) reporting on growth velocity and/or height with any ICS exposure compared to those without ICS exposure.

We excluded studies that recruited children where the diagnosis of asthma had not been established. We excluded crossover trials and studies that considered only oral corticosteroid use without reporting the effects of inhaled corticosteroids.

### Search Strategy

We initially searched MEDLINE and EMBASE in June 2013 using a broad strategy for a wide range of adverse effects, and we updated this with a more focused PubMed search in December 2014 (see S1 Appendix for search terms and restrictions). We also checked the bibliographies of included studies and existing systematic reviews for relevant articles.

### Study Selection

Two reviewers (MT and PB) independently, and in duplicate considered all titles and abstracts. At this point, we excluded reports that were clearly not RCTs or observational studies of ICS in asthma. We then retrieved full text versions of potentially relevant articles and carried out further screening to determine if the study objectives included the evaluation of growth. A third researcher (YKL) finalized the decision during discussion with the two reviewers.

### Study Characteristics and Data Extraction

We extracted data onto pre-formatted tables with details on study design, participants, definition of asthma, drug therapy (dose, device and frequency), and duration of follow-up. Two reviewers independently extracted data (MT and PB) on final adult height and growth velocity (cm per year). A third reviewer (YKL) corrected any discrepancies after rechecking the source papers.

### Risk of Bias Assessment

Two reviewers (MT and PB) independently assessed the reporting of blinding, allocation concealment, withdrawals and the loss to follow-up in RCTs. In accordance with recommendations on assessing adverse effects, we extracted information on participant selection, ascertainment of exposure and outcomes, and methods of addressing confounding in observational studies. [9] A third reviewer (YKL) adjudicated and made the final decision on discrepant items after rechecking the source papers.

We aimed to use a funnel plot to assess publication bias if there were >10 studies in the meta-analysis, with no significant heterogeneity seen. [10]

### Statistical Analysis

We pooled trial data using Review Manager (RevMan) version 5.3.2 (Nordic Cochrane Center, Copenhagen, Denmark). We used the inverse variance method to pool mean differences in growth velocity (cm per year) or final adult height (cm) between ICS users and non-users. We calculated the I^2^ statistic to for statistical heterogeneity with I^2^> 50% indicating a substantial level of heterogeneity. [11]

If a study had multiple arms involving different ICS doses, we attempted to analyse data on the licensed dose for children where available, otherwise we combined all the intervention arms together as recommended by the Cochrane Handbook. [12] For studies with more than one group of non-ICS users, we analysed data from the placebo arm (wherever possible) in preference to data from active comparators such as nedocromil or montelukast. If the study reported growth velocity values at different points over a number of years, we analysed the data based on the first year, but also recorded the overall change over the complete follow-up, as well as at different annual intervals. For studies that did not explicitly report growth velocity, we extracted data on the mean change in height from baseline to the 52 week follow-up. In accordance with the recommendations of the Cochrane Handbook, we imputed any standard deviations from 95% confidence intervals or p-values. [13]

### Subgroup Analysis

We aimed to conduct subgroup analysis where growth data were available for head to head comparisons of different treatment regimens (dose / duration), or for children of different ages within the same study.

We do not have a pre-registered protocol.

## Results

We screened 1882 potentially relevant articles, and finally included 23 studies in our systematic review (comprising twenty RCTs, [14–33] and three observational studies [34–36]). The process of study selection is shown in S1 Fig.

S1 and S2 Tables show the characteristics of the included RCTs, and the observational studies respectively. S3 and S4 Tables report on study validity and outcomes.

The study with the longest treatment duration was four years (CAMP—Childhood Asthma Management Programme), [24] while the remaining trials had ICS therapy for between 52–156 weeks. The ICS formulations reported in the trials included beclometasone, budesonide, ciclesonide, flunisolide, fluticasone, and mometasone. There was variation in the choice of control intervention, with placebo, nedocromil, montelukast, sodium cromoglicate being used in some trials. We identified three trials where budesonide and fluticasone were compared head-to-head, without any non-ICS control arm. [14, 18, 19]

The observational studies looked at narrow range of ICS users, primarily focusing on budesonide (two studies), [34, 35] whilst one study did not specify ICS formulation.[36]

### Study Validity

#### Randomized Controlled Trials (n = 20)

Nine of the RCTs reported an appropriate method of sequence generation, eleven provided details on how concealment of allocation was achieved whilst 13 reported the use of double blinding. (S2 Table). Ascertainment of height was usually done through stadiometry. S2 Table also shows that discontinuations and substantial losses to follow-up (particularly where height was not available at final timepoint) are major limitation affecting almost all the trials. The vast majority of trials had involvement of pharmaceutical industry sponsors.

#### Observational studies (n = 3)

Overall, we considered the studies to be at moderate to high risk of bias due to potentially confounded comparisons arising from the relatively limited matching and lack of multivariate adjustment.

### Outcomes: Growth Velocity

**Fig 1:**
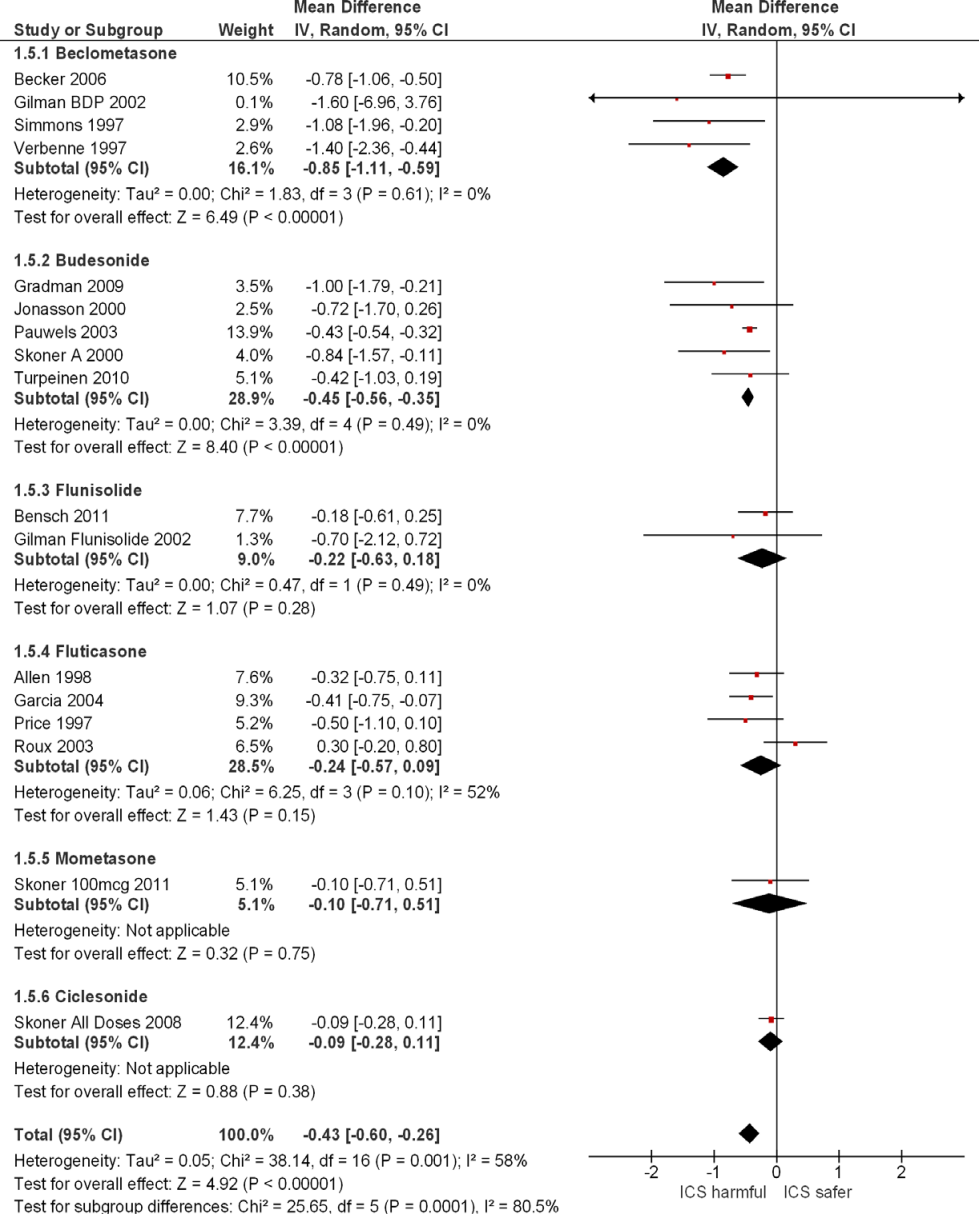
Growth Velocity in RCTS at 12 months follow-up.

Sixteen RCTs and one observational study reported on comparative difference in growth velocity (cm/year) in children. (Fig 1) Overall, ICS use was associated with significant reductions in growth velocity as compared to controls in RCTs (pooled Mean difference -0.48 cm/year^1^; 95% CI -0.66–0.29 cm/year; I^2^ = 48%). We demonstrated that agents such as beclometasone, budesonide, and fluticasone were all individually associated with significant reductions in growth velocity compared to non-users. However, there was only sparse data available for other formulations such as ciclesonide (one trial), [29] flunisolide (two trials), [17, 21] or mometasone (one trial), [30] and the broad confidence intervals reflect considerable uncertainty about treatment effects.

The magnitude of reduction in growth velocity in the meta-analysis of RCTs was similar to that seen with an observational study with 2.5 years’ follow-up in the Netherlands (Mean difference -0.44 cm/year; 95% CI -1.25 to 0.37 cm/year). [35] However, this study lacked power to detect a difference as there were only 66 children available for analysis.

### Outcomes: Final Adult Height

We identified one large long-term RCT of budesonide that captured final adult height in >90% of the originally enrolled children. [24] This trial found that four years of budesonide use was associated with a mean difference in final height of -1.2 cm (95% CI -1.9 − -0.5 cm) as compared to those on placebo.

**Fig 2:**
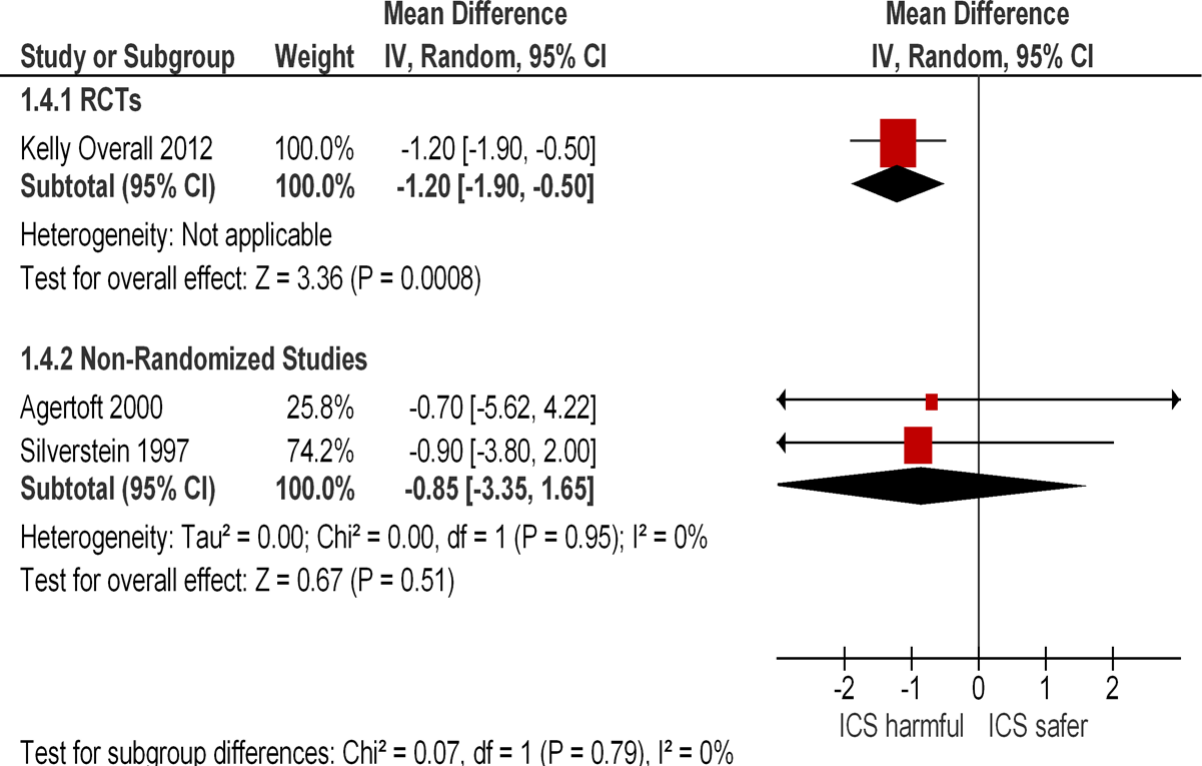
Final Adult Height, ICS users vs. non-users.

We identified two observational studies that reported reduction of between 0.7–0.9 cm in final adult height. [34, 36] However, the sample sizes were small (leading to imprecise estimates with broad confidence intervals) and a meta-analysis of the two observational studies showed a mean difference of -0.85cm (95% CI -3.35–1.65, I^2^ = 0%). (Fig 2)

### Subgroup Analysis Dose Response

**Fig 3:**
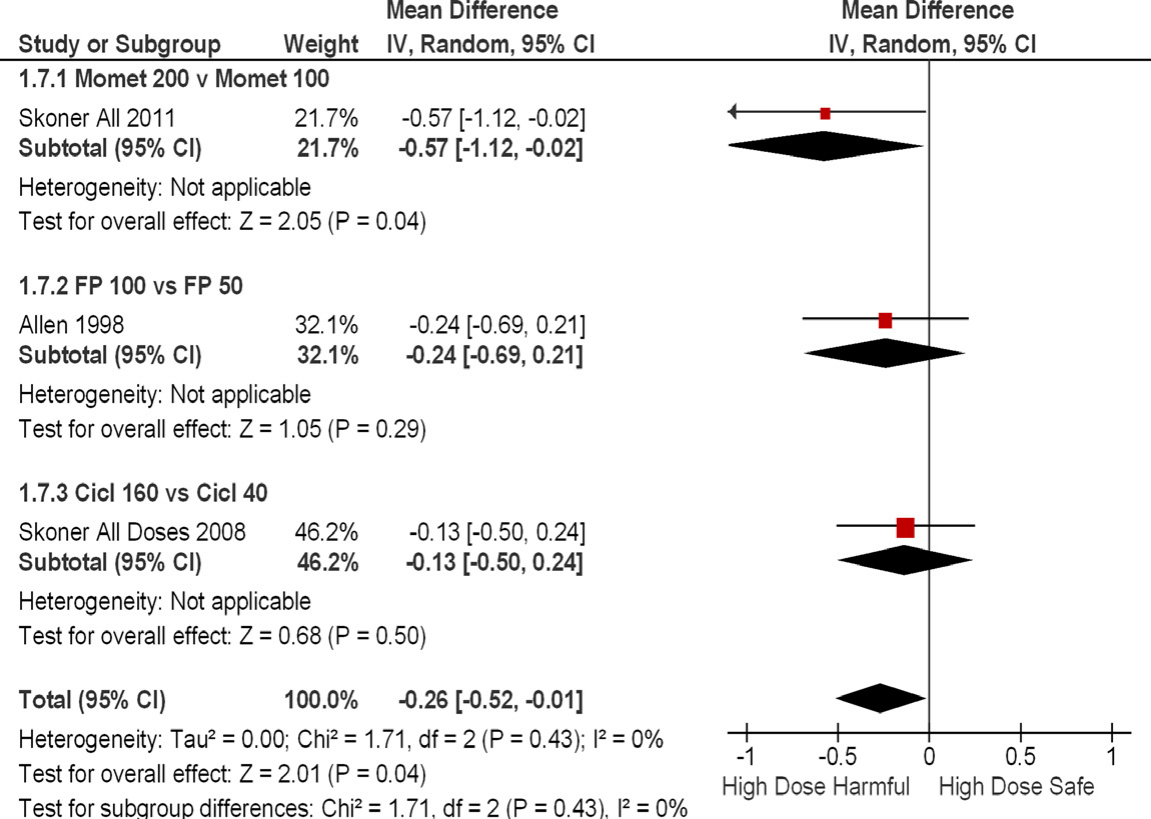
Mean Differences in Growth Velocity with Direct Comparison of Higher Dose vs. Lower Dose.

We identified 3 RCTS where we were able to conduct head to head comparisons of growth velocity between a higher dose and a lower dose of the same compound. [15, 29, 30] The trials were not powered to identify statistically significant differences in growth velocity with ascending ICS doses or different ICS formulations. However, we noted a consistent finding towards reduced growth velocities in the higher-dose arms relative to the lower-dose arms of the ICS, with a pooled estimate confirming greater harm (p = 0.04)with higher doses overall. (Fig 3).

### Subgroup Analysis Effect of Different ICS Compounds

Visual inspection of the Forest plot (Fig 1) suggests that magnitude of growth reduction may differ amongst the ICS compounds. When we conducted subgroup testing for differences between fluticasone and other ICS, we found that budesonide was not significantly worse (p = 0.15), whereas beclometasone had a significantly greater adverse impact (p = 0.007).

This was corroborated in a direct head-to-head comparison where there was a significant reduction in growth velocity of 0.91cm/year (95% CI 0.63–1.20) with beclometasone 400 mcg daily compared to fluticasone 400 mcg daily. [18] In contrast, we identified divergent findings in direct randomized trials comparing budesonide against fluticasone. Acun’s trial of budesonide 400 mcg vs. fluticasone 250 mcg showed better growth velocity with budesonide (+0.37 cm/year), [14] whereas Ferguson’s study of budesonide 400 mcg vs fluticasone 200 mcg reported significant adverse impact on growth velocity with budesonide of -0.9 cm/year (SE 0.19) compared to fluticasone. [19]

### Subgroup Analysis Change in Growth Velocity by Duration of Follow-Up

We identified findings in three RCTs where ICS appeared to have the greatest adverse impact on growth velocity in the first 12 months of the trial, whereas the reduction in growth velocity was less problematic at 24 or 36 months follow-up. [25, 27, 37] For instance, the large CAMP trial found a 1.7 cm/year reduction in growth velocity in the first 12 months for ICS versus control, but the difference in growth velocity was only about 0.1 cm at the 36 month follow-up. [37]

In contrast, a smaller trial did not demonstrate any variation in effect of ICS on growth velocity between the 12 and 24 months follow-up. [23]

### Subgroup Analysis Age of Participants

Three trials reported the impact of ICS on growth for children of different age groups. [23–25] Both Jonasson et al. and Pauwels et al. found that reduction in growth velocity with ICS was more marked in those age ≤11 years as compared to older children. [23, 25] The large CAMP trial found that when compared to controls, ICS reduced final adult height by -1.9 cm (95% CI -3.2 to -0.6 cm) in children entering the trial at age 5–8 years, whereas the reduction in final adult height was -0.5 cm (95% CI -1.7 to -0.6 cm) in those entering at age 9–13 years. [24]

### Reporting Biases

As there was substantial heterogeneity in the main analysis (Fig 1), we did not proceed to construct a funnel plot. The potential direction of reporting biases in the included studies remains unclear because we cannot judge conclusively if authors were more inclined to report significant adverse effects, or conversely, to play down any indication of harm. However, as mostof the studies involved commercial sponsors, we should be aware of the possibility of benefits being emphasized, whilst harms are presented in less detail.

## Discussion

Our meta-analysis of 23 studies confirms that long-term use >12 months of ICS is associated with a slight reduction in growth velocity and final adult height in children. The reliability of these results is supported by the concordance between the findings of the randomized and non-randomized studies in direction and magnitude of effect. Moreover, we also identified dose-responsiveness from direct randomized comparisons where lower doses of ICS were less harmful than higher doses. Subgroup data indicates that adverse effects on growth may be more prominent in younger children as opposed to older children. Equally, in trials where children were followed-up for 24–36 months, we found that the reduction in growth velocity appeared to be most prominent in the first year of therapy, and the magnitude of the adverse effect seemed less problematic with time. This would tie in with the relatively limited diminution in final adult height, whereas we would have expected a cumulative decrement of several centimeters if children had continued to fall behind by 0.5 cm for every cumulative year of ICS therapy.

The effect size should be interpreted in the context of the average adult height of the population. For instance, in England, the average adult height is 175.3 cm for men, and 161.6 cm for women. [38] In an adult male, the reduction of around 1.2 cm would represent only a 0.68% absolute diminution (from 175.3 cm to 174.1 cm). This might be considered as an almost imperceptible or clinically insignificant change, particularly when weighted up against of the proven benefits of ICS therapy. We should be conscious too that the reported 1.2 cm diminution probably represents a worst case scenario where participants in the RCT were using Budesonide 400 mcg daily continuously for a three year period, whereas real-world have their doses titrated up and down according to disease severity. [39] In addition, adherence to therapy, and therefore adverse effects of medication, is likely to be greater in a RCT than a real-world setting.

There are a number of potential explanations for these findings. With longer durations of follow-up, the initial adverse consequences on growth velocity may become less problematic if the child has better-controlled asthma (thus enabling greater physical activity) and fewer exacerbations (associated with reduced need for acute short-courses of oral corticosteroids). Moreover, Pedersen has pointed out that children have different growth phases according to age. [6] Hence, susceptibility to the adverse effects of ICS seems to be less of a problem in those above the age of 10 years or more, which is a consistent finding in a number of trials.

Our findings are similar to those of recent Cochrane systematic reviews that have focused principally on data from RCTs. [40, 41] In a meta-analysis of 15 RCTs, Zhang et al. reported that ICS use was associated with significantly reduced growth velocity, mean difference of -0.48 cm/year as compared to controls. [41] In contrast to our review, Zhang et al. had selection criteria that included RCTs of <12 months, but excluded observational studies. Pruteanu et al. conducted a meta-analysis which demonstrated that higher ICS doses were associated with greater reductions in growth velocity (a finding that is similar to ours), but they were unable to identify significant differences amongst the ICS compounds. [40]

There are a number of limitations to our systematic review. Owing to the wide variety of possible combinations of drug compounds, inhaler devices, and daily doses, we felt that it would be scientifically inappropriate to draw conclusions through confounded indirect comparisons on dose or drug compound. Instead, we have limited our subgroup analyses to trials that performed direct comparisons of dose or different ICS, and we do not have consistent evidence that any specific compound is safer. The studies in our review date back 10–20 years and the findings may be less applicable to current-day children in the face of better nutrition and innovations in asthma management.

There are also a number of important limitations relating to validity of the primary studies. Owing to the inherent difficulties in measuring long-term adverse events, there is potential for considerable risk of bias (particularly from attrition) within this dataset. Moreover, the vast majority of the trials were sponsored by the pharmaceutical industry, and the study methodology (e.g. when choosing interventional dose and device) may have been designed towards obtaining favourable results for the sponsored product. Equally, we should be wary of potential reporting biases where positive findings are emphasized whereas negative ones are downplayed. This diversity amongst trial sponsors may have contributed towards the divergent findings and increased heterogeneity in results.

Lack of information and inaccurate interpretation of the benefits and harms of ICS may hinder medication adherence in patients with asthma. [42] Although the evidence may be imperfect, our systematic review helps to address some of the concerns and uncertainty surrounding the exact magnitude of growth reduction with ICS use. There is some indication of potential differences between ICS formulations, with beclometasone possibly having a greater adverse impact on growth. Prescribers and patients should aim for the lowest effective dose, particularly if initiating ICS therapy for patients in the younger age groups who may be more susceptible to adverse effects.

## Supporting Information

S1 Appendix Search Strategy. (DOCX)

S1 Fig Flow Diagram of process of study selection. (PDF)

S1 PRISMA Checklist (DOC)

S1 Table Characteristics of randomized controlled trials included in the analysis of growth. (DOCX)

S2 Table Characteristics of observational studies. (DOCX)

S3 Table Risk of bias assessment and growth outcomes in RCTs of inhaled corticosteroids. (DOCX)

S4 Table Study Validity and Growth Outcomes in Observational Studies. (DOCX)
