# Supplementary material for: Impact of Inhaled Corticosteroids on Growth in Children with Asthma: Systematic Review and Meta-Analysis
Source: PLoS One. 2015 Jul 20;10(7):e0133428. doi: 10.1371/journal.pone.0133428 (PMC4507851; doi:10.1371/journal.pone.0133428)
Supplement: S1 Appendix — (DOCX) [file pone.0133428.s001.docx]

Ovid SP search (EMBASE and MEDLINE) from database inception to July 2013

Limited to English, Human, with Abstract

Disease term: asthma

AND

Intervention term: (beclometasone OR beclomethasone OR fluticasone OR budesonide OR mometasone OR triamcinolone OR inhaled-corticosteroid OR inhaled-corticosteroids OR ciclesonide OR inhaled-steroid or inhaled-glucocorticoid).mp

AND

Adverse effect terms such as: (fracture$ OR cataract$ or glaucoma$ OR growth OR height OR stature OR pituitary OR hypothalamic OR diabetes OR glucose).mp

PubMed Update Dec 2014

("Anti-Asthmatic Agents/adverse effects"[MeSH Terms] OR "Administration, Inhalation"[MeSH Terms] OR inhaled-corticosteroid[All Fields] OR inhaled-glucocorticoid[All Fields]) AND (("growth and development"[Subheading] OR ("growth"[All Fields] AND "development"[All Fields]) OR "growth and development"[All Fields] OR "growth"[All Fields] OR "growth"[MeSH Terms]) OR height[All Fields] AND ("asthma"[MeSH Terms] OR "asthma"[All Fields])
