# Supplementary figures and images for: Impact of Inhaled Corticosteroids on Growth in Children with Asthma: Systematic Review and Meta-Analysis
Source: PLoS One. 2015 Jul 20;10(7):e0133428. doi: 10.1371/journal.pone.0133428 (PMC4507851; doi:10.1371/journal.pone.0133428)

**Figure 1. Flow Diagram of Study Selection**

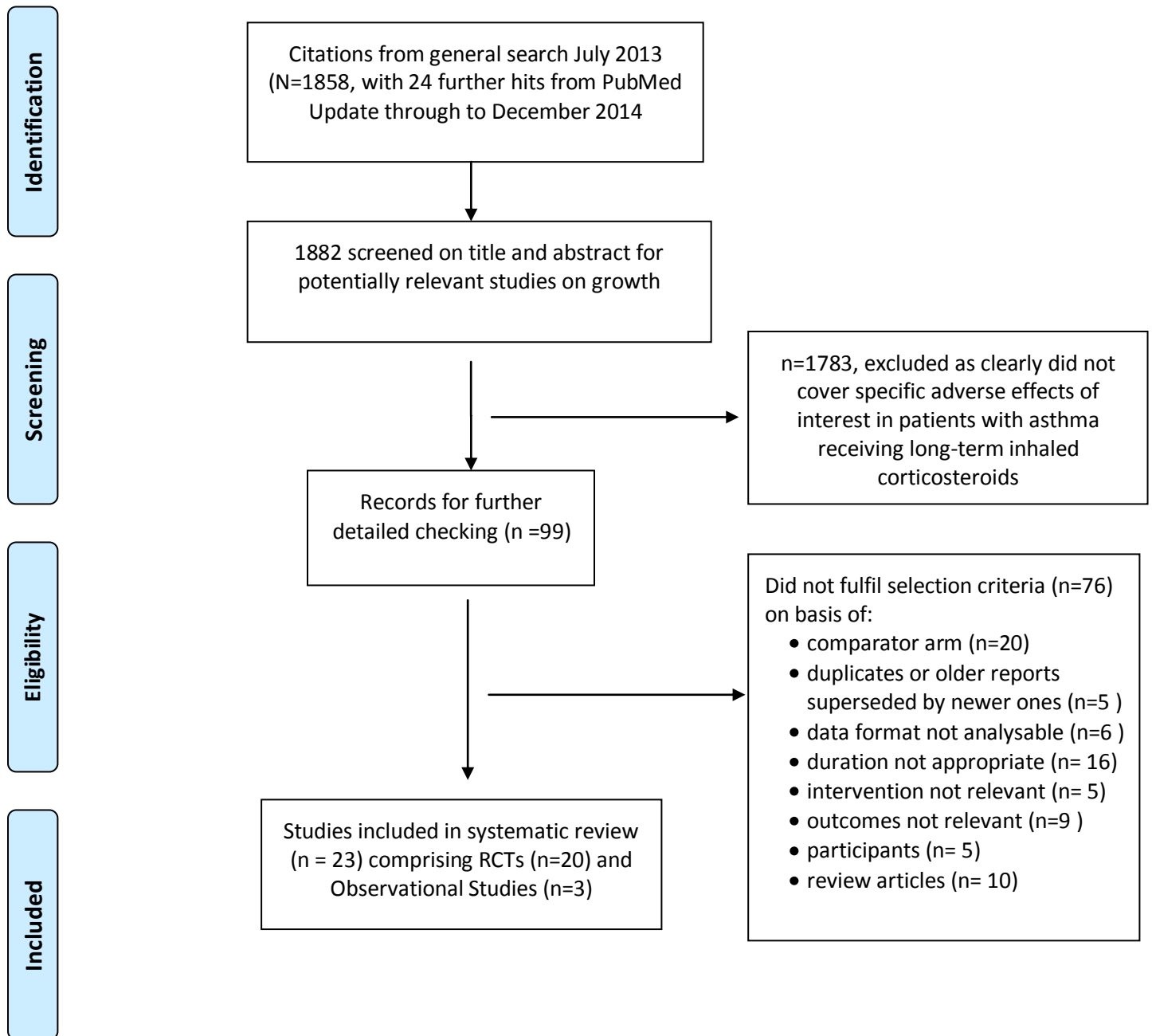

Supplement: S1 Fig — (PDF) [file pone.0133428.s002.pdf]
