# Supplementary material for: Impact of Inhaled Corticosteroids on Growth in Children with Asthma: Systematic Review and Meta-Analysis
Source: PLoS One. 2015 Jul 20;10(7):e0133428. doi: 10.1371/journal.pone.0133428 (PMC4507851; doi:10.1371/journal.pone.0133428)
Supplement: S1 Table — (DOCX) [file pone.0133428.s004.docx]

S1 Table. Characteristics of randomized controlled trials included in the analysis of growth

| **Source** | **Location and Source of Funding** | **Treatment**  **Duration,**  **Wks** | **Asthma Criteria** | **Drug and Device** | **Male,**  **%** | **Mean Age,**  **Years (SD)** | **Mean % Predicted**  **FEV1 (SD)** | **Prior**  **ICS use**  **(%)** |
| --- | --- | --- | --- | --- | --- | --- | --- | --- |
| Acun 2005 (14) | Turkey, no details on funding | 52 weeks | Moderate persisting asthma | Bud 400mcg/day (Pulmicort MDI) | 20 | 6.62+/-1.98 | NR | NR |
|  |  |  |  | FP 250mcg/day (Flixotide MDI) | 31 | 7.1+/-2.42 | NR | NR |
| Allen 1998 (15) | Nineteen clinical centers in USA, pharmaceutical company funding | 52 weeks | American Thoracic Society criteria. Persistent asthma ≥3 months and FEV1 of ≥60% predicted. | FP 50 μg (Diskhaler DPI) | 62(73%) | 8.1 ± 0.2 | 89 ± 1 | 39 (36) |
|  |  |  |  | FP 100 μg (Diskhaler DPI) | 72 (75%) | 8.1 ± 0.2 | 88 ± 1 | 44 (41) |
|  |  |  |  | Placebo (n=87): | 67 (77%) | 7.9 ± 0.2 | 88 ± 2 | 39 (37) |
| Becker 2006 (16) | Multicentre study (30 centres worldwide), pharmaceutical company funding | 56 weeks | Mild asthma | Montelukast 5mg | 73 (61%) | 7.50 +/- 0.74 | 92.3 +/-16.3 | NR |
|  |  |  |  | BDP 200mcg (MDI) | 80 (67%) | 7.57 +/- 0.76 | 91.3 +/-15.8 | NR |
|  |  |  |  | Placebo | 79 (65%) | 7.68 +/- 0.82 | 92 +/- 16.4 | NR |
| Bensch 2011 (17) | 45 centres in USA. pharmaceutical company funding | 52 weeks (1 year) | FEV1 ≥75 before salbutamol. At least 12% ↑FEV1 after salbutamol. peak flow diurnal variability ≥20% for ≥4 days a week | Flunisolide HFA (85 mcg bd) | 63 (59.4) | 6.5 (1.57) | 96.4 +/- 12.45 | NR |
|  |  |  |  | Placebo | 66 (58.9) | 6.4 (1.57) | 95.9 +/- 13.34 | NR |
| De Benedicts 2001 (18) | Holland, Hungary, Italy, Poland Argentina, Chile, South Africa, pharmaceutical company funding | 52 weeks (12 months) | PEF ≤ 85% of maximum achievable response after salbutamol. Asthma symptom score of ≥ 1X daily on ≥4 days of the week. | FP 200 mcg twice daily (Diskhaler DPI) (n-170) | 113 | 7.6 (1.7) | NR | 162 (95.3) |
|  |  |  |  | BDP 200 mcg twice daily (Diskhaler DPI) | 135 | 7.6 (2.0) | NR | 166 (96.0) |
| Ferguson 2006 (19) | Multicentre – 35 centres in 11 countries, 1999-2001, pharmaceutical company funding | 52 | Age 6-9 years persistent asthma ≥ 6 months; FEV1 ≥ 60% predicted; PEFR↑ of ≥ 15% after salbutamol. Exclusions: oral corticosteroids on > 2 occasions or > 12 days or > 210 mg prednisolone past 6 months; known growth disorder or glaucoma/cataracts. | FP 100mcg twice daily (Diskus DPI) | 77 (68) | 7.2 (1.0) | 90.2 (84) | 29 (25) |
|  |  |  |  | BUD 200mcg twice daily (Turbuhaler DPI) | 83 (70) | 7.4 (1.0) | 92.3 (17.9) | 25 (21) |
| Garcia 2005 (20) | International study at 104 sites in 24 countries, pharmaceutical company funding | 52 weeks | Mild persistent asthma (GINA guidelines) | Montelukast 5 mg | 321 (64.8) | 9 (6-14) | 86.8 (34.2-129) | 86 (17.8) |
|  |  |  |  | Fluticasone 100 mcg twice daily (MDI) | 292 (58.5) | 9 (5-15) | 87.7 (51.8-125) | 51 (10.5) |
| Gillman 2002 (21) | 24 centres in US, pharmaceutical company funding | 52 weeks | Mild to moderate asthma | Flunisolide HFA 340mcg daily | 91 (59.9) | 8.0 +/- 2.3 | NR | NR |
|  |  |  |  | BDP 336mcg daily (MDI) | 29 (74.4) | 8.9 +/- 1.5 | NR | NR |
|  |  |  |  | Cromolyn sodium 6,400mcg | 25 (56.8) | 8.6 +/- 1.5 | NR | NR |
| Gradman and Wolthers 2010 (22) | Secondary care pediatric outpatient clinic, Denmark, mixture of charity and industry funding | 52 weeks | mild persistent asthma according to the GINA guidelines | Bud 200 mcg (Pulairmax DPI) | 16/25 | 9.2 (5-11) | 88 (12) | NR |
|  |  |  |  | Montelukast 5 mg. | 21/27 | 8.8 (5-11) | 86 (11) | NR |
| Jonasson 2000 (23) | UllevaÊl Hospital, Oslo, pharmaceutical company funding | 117 weeks (27 months) | Mild asthma | BUD 100 mcg Turbuhaler DPI | 23/28 | 9.5 | NR | NR |
|  |  |  |  | BUD 200 mcg Turbuhaler DPI | 17/32 | 10.0 | NR | NR |
|  |  |  |  | BUD 100 mcg twice daily Turbuhaler DPI | 18/28 | 10.2 | NR | NR |
|  |  |  |  | Placebo | 22/34 | 9.4 | NR | NR |
| Kelly 2012 (37) (24) | Multicentre, 7 centres in US, 1993 -1999, government funding | >208 weeks | Mild-to-moderate asthma by symptoms or inhaled bronchodilator ≥twice weekly or daily medication for asthma. Airway methacholine challenge test. | Bud 200 twice daily Turbuhaler DPI | 181 (58%) | 9.0 ± 2.1 | 93.6 ± 14.4 | 126 (40.5%) |
|  |  |  |  | nedocromil 8 mg twice daily (Tilade) | 206  (66%) | 8.8 ± 2.1 | 93.4 ± 14.5 | 114 (36.5%) |
|  |  |  |  | placebo | 234  (56 %) | 9.0 ± 2.2 | 94.2 ± 14.0 | 150 (35.9%) |
| Pauwels 2003 (25) | 32 countries, pharmaceutical company funding | 156 weeks – 3 years | Mild persistent asthma. | BUD 400 mcg (200 μg for age < 11 years), Turbuhaler DPI | unclear | 24 (15) | 86.3 (13.9) | NR |
|  |  |  |  | Placebo |  | 24 (15) | 86.3 (13.9) | NR |
| Price 1997 (26) | 15 centres in United Kingdom, asthma clinics and primary care, pharmaceutical company funding | 52 weeks | Aged 4 to 10 years, with mild asthma. history of recurrent episodes of wheeze and cough. | FP 50 mcg bd Diskhaler | n = 33 (63%) | 6.0 ± 1.4 | Mean % predicted PEF: 84.6 ± 15.9 | No inhaled therapy within the last year |
|  |  |  |  | DSCG 20 mg qds  DPI | N = 42 (60%) | 6.4 ± 1.6 | Mean % predicted PEF: 87.3 ± 15.8 | No inhaled therapy within the last year |
| Roux 2003 (27) | 52 respiratory specialist clinics in France, pharmaceutical company funding | 104 weeks | Exacerbations ≥1X/ week but <1X daily; or chronic symptoms requiring daily treatment. Fulfilling: (1) FEV1 or PEF ≥80% predicted; (2) reversibility ≥15% (3) daily variability PEF 20%-30% ≥ 2 days, or salbutamol use >3 times previous week, or nocturnal symptoms ≥2X during run-in. | FP 100 mcg bd (n=87)  Diskus/ Accuhaler DPI | 64% | 9.1 ± 2.5 | 88.9 ± 12.4 | NR |
|  |  |  |  | Nedocromil sodium 4mg bd (n=87)  MDI | 66% | 9.4 ± 2.4 | 88.5 ± 14.1 | NR |
| Simons 1997 (28) | Multicentre. Winnipeg, Canada. 1992 to 1995, pharmaceutical company funding | 52 weeks | Clinically stable, persistent asthma < one month of prior inhaled or oral glucocorticoid use for asthma. FEV1 > 70% after bronchodilator withheld for 6 hours; 10% increase in FEV1 30 minutes after inhalation of 400 μg salbutamol. | BDP 200 mcg bd  Diskhaler DPI | 59% | 9.6 ± 2.6 | 92 ± 13 | None within past 3 months |
|  |  |  |  | Salmeterol xinafoate 50 mcg bd  Diskhaler DPI | 60% | 8.8 ± 2.1 | 95 ± 13 | None within past 3 months |
|  |  |  |  | Placebo bd (lactose)  *DPI* | 55% | 9.5 ± 2.4 | 96 ± 16 | None within past 3 months |
| Skoner et al 2000 (31) | 3 open-label, multicentre (26 centres) in the United States, pharmaceutical company funding | 52 weeks | Boys and girls aged 6 months to 8 years, with persistent asthma, recruited from 3 multicentre 12-week RCTs, enrolled into open-lable randomized extension.  Study A – no prior ICS intervention | *BUD Inhalation Suspension,(n = 182):*  Initially 0.5 mg once daily | 125 (68.7%) | 58.4 ± 26.2 months | FEV1: 81.15 ± 19.19 | 100% |
|  |  |  |  | *Control (n = 90):*  Cromolyn sodium 79%, albuterol 52%, nedocromil 8%, theophylline 6%. | 57 (63.3%) | 60.4 ± 26.3 months | FEV1: 82.62 ± 19.42 | NR |
| Skoner et al 2008 (29) | 85 centres in 4 countries, US and South America, 2000 to 2004, pharmaceutical company funding | 52 weeks | Children (aged 5.0 to 7.5 years in females, 5.0 to 8.5 years in males), with mild persistent asthma for ≥ 3 months before screening and FEV of ≥ 80% predicted after ≥ 4-hour albuterol withhold. | Ciclesonide 40 mcg qds MDI | n = 150 67.9% | 6.6 ± 0.97 | 96.42 ± 12.29 | 19.5 % |
|  |  |  |  | Ciclesonide 160 mcg qds MDI | n = 147  67.1% | 6.7 ± 0.93; | 96.07 ± 11.26 | 21.0 % |
|  |  |  |  | Placebo once daily | n = 147 66.5% | 6.7 ± 0.95; | 95.98 ± 11.39 | 19.0 % |
| Skoner et al 2011 (30) | Multicentre study, United States, pharmaceutical company funding | 52 weeks treatment period followed by 3 months follow-up period | Age 4 to 9 years with persistent asthma ≥ 6 months.  FEV1 of > 75% of predicted screening visit and baseline visit, when all restricted medications had been withheld. Increase in absolute FEV1 of at least 12% after reversibility testing at screening visit or historically within past 12 months. | MF-DPI) 100 mcg once daily. | 34  (71 %) | 6.4 years | 87.2 (range 61.0 to 115.3) | NR |
|  |  |  |  | MF-DPI) 100 mcg bd | 28  (64 %) | 6.3 years | 89.3 (range 74.9 to 111.3) | NR |
|  |  |  |  | MF-DPI) 200 mcg once daily | 33  (66 %) | 6.6 years | 89.5 (range 47.5 to 112.1) | NR |
|  |  |  |  | Placebo | n = 36  80 % | 6.6 years | 85.4 (range 61.4 to 115.3) | NR |
| Turpeinen et al 2010 (32) | Helsinki University Hospital, Finland, pharmaceutical company and hospital funding | 72 weeks | “Newly detected mild asthma”  Excluded if history of inhaled, nasal or oral corticosteroid use in the previous 2 months before enrollment. | Continuous BUD (n=50) Turbuhaler 400 mcg bd 1^st^ month, then 200 mcg bd 2^nd^ – 6th month, then 100 μg bd 7th – 18th month | 60 | 6.9 | Not reported | NR |
|  |  |  |  | BUD/Placebo (n=44) Turbuhaler, 400 μg bd 1^st^ month, then 200 mcg bd 2^nd^ to 6^th^ month, then placebo 7^th^ – 18^th^ month. | 66 | 6.7 | NR | NR |
|  |  |  |  | DSCG MDI – 10mg tds for 1^st^ to 18^th^ month (unblinded) (n=42) | 50 | 7.0 | NR | NR |
| Verberne et al 1997 (33) | Outpatient paediatric clinics, multicenter, Netherlands, 1992 – 1994, pharmaceutical company funding | 54weeks. | 67 children aged 6 to 16 years with mild to moderate asthma according to American Thoracic Society criteria. | Salmeterol xinafoate 50 mcg bd *DPI* | n = 23 (72%) | 10.6 ± 2.9 | 85.6 ± 15.0 | n = 5 (16%) |
|  |  |  |  | Beclomethasone dipropionate 200 mcg bd *DPI* | n = 22 (63%) | 10.5 ± 2.3 | 86.3 ± 13.6 | n = 6 (17%) |

BDP: beclometasone diproprionate; Bud: budesonide; DSCG: sodium cromoglicate; DPI: dry powder inhaler; FP: fluticasone propionate; MDI: Metered dose inhaler; MF: mometasone furoate. NR: Not Reported PEF: Mean morning peak expiratory flow
