# Supplementary material for: Impact of Inhaled Corticosteroids on Growth in Children with Asthma: Systematic Review and Meta-Analysis
Source: PLoS One. 2015 Jul 20;10(7):e0133428. doi: 10.1371/journal.pone.0133428 (PMC4507851; doi:10.1371/journal.pone.0133428)
Supplement: S2 Table — (DOCX) [file pone.0133428.s005.docx]

**S2 Table: Characteristics of observational studies**

| **Study** | **Design** | **Adverse Effects Measured** | **Data source and Number of Patients** | **Selection of patients: Asthma definition & Patient Characteristics (or Selection of Cases and Controls)** | **Type of ICS** |
| --- | --- | --- | --- | --- | --- |
| **Agertoft & Pedersen 2000** **(34)** | Prospective long-term study | Final adult height | Kolding hospital, Denmark.  142 ICS users that reached adult age.  18 controls: not on ICS. | Paediatric clinic, 6 monthly attendance for 1-2 years  Budesonide group – 86 boys/56 girls. Age at the start of treatment (mean) 8.7, at attainment of adult age 18.0.  Control group – 11 boys/7 girls. Age at adult height (mean) 18.5. | Budesonide |
| **Merkus 1993** **(35)** | Case-control | Growth rate | 40 asthmatic teenagers in outpatient respiratory clinics – Netherlands (1987-1991). | Controls: 621 secondary school children with no asthma history, matched for gender, age, standing height and duration of follow up.  Salbutamol +Budesonide = 22; Controls = 44 | Budesonide |
| **Silverstein 1997** **(36)** | Retrospective cohort | Attained adult height | 153 residents with asthma Rochester, Minnesota, (1964 to 1987) and age- and sex-matched non- asthmatic. | 78 women (51%) and 75 men (49%). Mean age at onset of asthma was 6.1+/- 4.8 years, mean age at first glucocorticoid exposure was 12.5 +/- 3.9 years | Not specified |
