# Supplementary material for: Impact of Inhaled Corticosteroids on Growth in Children with Asthma: Systematic Review and Meta-Analysis
Source: PLoS One. 2015 Jul 20;10(7):e0133428. doi: 10.1371/journal.pone.0133428 (PMC4507851; doi:10.1371/journal.pone.0133428)
Supplement: S3 Table — (DOCX) [file pone.0133428.s006.docx]

S3 Table. Risk of bias assessment and growth outcomes in RCTs of inhaled corticosteroids

| **Source** | **Sequence generation** | **Allocation Concealment** | **Blinding** | **Height monitoring** | **Drug (n)** | **Height (cm) or Mean Growth Velocity (GV in cm/year)** | **Discontinued, No. (%)** | **Missing outcome data, No (%)** |
| --- | --- | --- | --- | --- | --- | --- | --- | --- |
| Acun 2005(14) | Unsure | Unclear | Open-label | Growth measured at each visit | BUD (20) | GV: 6.37 ± 3.09 | NR | NR |
|  |  |  |  |  | FP (31) | GV: 6.0 ± 0.94 | NR | NR |
| Allen 1998(15) | Unclear | Unclear | Double blind | Growth measured monthly | FP 50 μg (111) | GV: 5.91 ± 0.16 | 2% | NR |
|  |  |  |  |  | FP 100 μg (108) | GV: 5.67 ± 0.13 | 4% | NR |
|  |  |  |  |  | Placebo (106) | GV: 6.10 ± 0.17 | 23% | NR |
| Becker 2006(16) | Adequate | Adequate | Double blind | Calibrated stadiometer at each visit, staff undertook special training | Montelukast (120) | GV: : 5.67 (95% CI 5.46 - 5.88) | 11 | NR |
|  |  |  |  |  | BDP (119) | GV: 4.86 (95% CI: 4.64 - 5.08) | 11 | NR |
|  |  |  |  |  | Placebo (121) | GV: :5.64 (95% CI 5.42 - 5.86)  Difference in means for BDP vs placebo:  -0.78 cm (95% CI: -1.06 to – 0.49 cm) | 13 | NR |
| Bensch 2011(17) | Unclear | Unclear | Double-blind | Height measured in triplicate with stadiometer. | Flunisolide (106) | GV: 6.01 ± 1.84 | 41 | NR |
|  |  |  |  |  | Placebo (112) | GV: 6.19 ± 1.30 | 38 | NR |
| De Benedicts 2001(18) | Adequate | Adequate | Double-blind | Growth velocity was measured by means of stadiometry. | FP (170) | GV5.01 ± 0.14 | NR | 33 |
|  |  |  |  |  | BD (173) | GV4.10 ± 0.15 | NR | 33 |
| Ferguson 2006(19) | Adequate | Adequate | Double blind | Measurements in triplicate with Harpenden stadiometer by same researcher wherever possible. | FP ( 114) | Adjusted GV5.5  (SE 1.3) | 12 | 2 |
|  |  |  |  |  | BUD ( 119) | Adjusted GV4.6 (SE 1.5) | 19 | 2 |
| Garcia 2006(20) | Adequate | Unclear | Double- blind | Height measured with standard office stadiometer, and was recorded as average of 2 separate measurements. | Montelukast ( 495) | Difference in GV fluticasone vs. montelukast:  -0.41 (95% CI -0.75 to 0.07 ) | 36 (7.3%) | 6 (1.2%) |
|  |  |  |  |  | Fluticasone (499) |  | 33 (6.6%) | 5 (1.0%) |
| Gillman 2002(21) | Unclear | Unclear | Open label | Height measured by stadiometry. | Flunisolide ( 152) | GV: 6.2 ± 2.9 | 1 (2.6%) | NR |
|  |  |  |  |  | BDP ( 39) | GV: 5.1±1.9 | 9 (5.9%) | NR |
|  |  |  |  |  | DSCG (44) | GV: 6.2 ± 2.3 | 3 (6.8%) | NR |
| Gradman and Wolthers 2010(22) | Unclear | Unclear | Open-label | Height measured in triplicate with Harpenden stadiometer. | BUD (25) | GV: 5.51 ± 1.35 | NR | NR |
|  |  |  |  |  | Montelukast (27) | GV: 6.51 ± 1.36  Difference between Bud and Montelukast: -1.00 (95% CI: -0.20 - -1.79) cm | NR | NR |
| Jonasson 2000(23) | Unclear | Unclear | Double-blind | Height measured with stadiometer. Three trained persons carried out all height measurements during the study. | BUD 100 mcg daily (28) | GV: 5.38 ± 1.82 cm  Difference in GV between budesonide and placebo groups in second year:  0.72 (95% CI -0.26 - 1.70) | 6 | NR |
|  |  |  |  |  | BUD 200 mcg daily  (32) |  | 8 | NR |
|  |  |  |  |  | BUD 100 mcg bd (28) |  | 7 | NR |
|  |  |  |  |  | Placebo (34) | GV: 6.17 ± 1.13 | 13 | NR |
| Kelly 2012(24, 37) | Adequate | Adequate | Double-blind | Height measured every 6 months during the initial 4.5 years of observational follow-up and 1-2 times a year next 8 years. Adult height at a mean (±SD) age of 24.9±2.7 years. Trained technician used stadiometer to measure adult height for 943 of original 1041 participants. | Nedocromil ( 312) |  | NR | 27 |
|  |  |  |  |  | BUD ( 311) | Mean difference in final adult height, Bud vs. placebo-1.2cm (95% CI -1.9 - -0.5 cm)  Age at entry:  5-8 yrs: −1.9 (−3.2 to −0.6) cm  9-13 yrs: −0.5 (−1.7 to 0.6) cm | NR | 30 |
|  |  |  |  |  | Placebo (418) |  | NR | 41 |
| Pauwels 2003(25) | Adequate | Adequate. | Double-blind | Follow-up visits at 6 and 12 weeks after randomization, then every 3 months for 3 years. Height measured to nearest cm at each visit. | BUD (3,642 overall, but number of children not stated) | Mean difference GV Bud vs. placebo  age 5 – 15 years:-0.43 (95% CI -0.54 to -0.32) | 990 | 105 |
|  |  |  |  |  | Placebo (3,599 overall, but number of children not stated) |  | 1020 | 106 |
| Price 1997(26) | Unclear | Unclear | Open-label | Three measurements using Holtain stadiometer at each visit, using same equipment and same person whenever possible. Mean height velocity (adjusted for age and gender) | FP 50 mcg bd  (52) | **GV:** 6.0  Mean difference FP vs. control: -0.5cm/year  95% CI: -1.0 to +0.1 | 16 (31%) | 18 (35%) |
|  |  |  |  |  | DSCG 20mg four times daily ( 70) | **GV:** 6.5 | 43 (61%) | 44 (63%) |
| Roux 2003(27) | Adequate | Adequate. | Open label | Height measured at end of run-in, then 12 and 24 months. Growth velocity expressed in cm/year, adjusted for age, gender and baseline height. | Flu 200 mcg /day (87) | **GV:**:6.1 at 24 months follow-up  FP vs. control treatment difference -0.3 cm, 95% CI: -0.2 to + 0.8 cm/year | 13 (15%) | 2 (2%) |
|  |  |  |  |  | NS 8mg/day (87) | **GV:** 5.8  at 24 months | 28 (32%) | 5 (6%) |
| Simons 1997(28) | Unclear | Unclear | Double-blind | Height measured by same trained observer at each site, using calibrated stadiometer at most sites. | BDP200 mcg bd  (81) | **GV:** 3.96 | NR | 14 (17%) |
|  |  |  |  |  | Salm 50 mcg bd  (80) | **GV:** 5.40 | NR | 22 (28%) |
|  |  |  |  |  | Placebo bd (80) | **GV:** 5.04 | NR | 25 (31%) |
| Skoner 2000(31) | Unclear | Unclear | Open-label | Height measured by stadiometry, same professional doing stadiometric measurements. | BUD (182) | **GV:***:*6.55 ± 2.08 | NR | 32 |
|  |  |  |  |  | Controls: (90) | **GV:** 7.39 ± 2.51 | NR | 32 |
| Skoner2008(29) | Adequate  . | Adequate | Double-blind | Height measurements by trained technician using Harpenden stadiometer. Median of 4 acceptable serial measurements used. Identical stadiometers, calibrated within 4 hours of each measurement, same technician used at each visit when possible. | Ciclesonide 40 mcg once daily (221) | **GV:** 5.73 ± 0.08; 95% CI: 5.57 to 5.89.  Mean (SD) difference in GV versus placebo: -0.02 ± 0.09; 95% CI: -0.19 to +0.16. | n = 40 (18.1%) | 29 (13%) |
|  |  |  |  |  | Ciclesonide 160 mcg daily (219) | **GV:**: 5.60 ± 0.08; 95% CI: 5.44 to 5.76  2) Mean (SD) difference in GV versus placebo: -0.15 ± 0.09; 95% CI: -0.33 to +0.03 | n = 31 (14.2%) | 46 (21%) |
|  |  |  |  |  | Placebo (221) | **GV:** 5.75 ± 0.08; 95% CI: 5.59 ± 5.90 | n = 40 (18.1%) | 33 (15%) |
| Skoner 2011(30) | Unclear | Unclear | Double-blind | Measurements at each visit using Harpenden stadiometer. With mean of 3 values recorded. Same study personnel for given subject throughout study at same time of day for each visit. | MF-DPI 100 mcg once daily.  n = 48 at baseline | *1) LRS model (n = 48):*  **GV:** 6.42  Mean change in GV from placebo (SE): -0.10 ± 0.31 | n = 10 (21%) | 1 (2%) |
|  |  |  |  |  | MF-DPI 100 mcg bd  n = 44 at baseline | *1) LRS model (n = 42):*  **GV:** 5.88  Mean change in GV from placebo (SE): -0.64 ± 0.39 | n = 11 (25%) | 2 (5%) |
|  |  |  |  |  | MF-DPI 200 mcg once daily,  n = 50 at baseline | *1) LRS model (n = 49):*  **GV:** 5.82  Mean change in GV from placebo (SE): -0.70 ± 0.29 | n = 16 (32%) | 4 (8%) |
|  |  |  |  |  | Placebo  n = 45 at baseline | *1) LRS model (n = 45):*  **GV:** 6.52 | n = 15 (33%) | 6 (13%) |
| Turpeinen 2010(32) | Unclear | Unclear | DSCG arm open-label | Height recorded with a stadiometer following standardized procedure. Median value of three measurements recorded. | Initial BUD (58)  Final BUD (50) | *1)(a)Increment in height over 18 months:*7.75 cm; 95% CI: +7.34 to +8.17  *(b) compared with BUD/placebo*:  -0.42 cm; 95% CI: -1.03 to +0.18  *(c) compared with DSCG:*  -1.05 cm; 95% CI: -1.66 to -0.44 | n = 6 (10%) | NR |
|  |  |  |  |  | Initial BUD/placebo (58)  Final BUD/placebo (44) | *1)(a)Increment in height over 18 months:* 8.18 cm; 95% CI: +7.73 to +8.63  *(b) compared with DSCG:*  -0.63 cm; 95% CI: -1.26 to +0.01 | n = 12 (21%) | NR |
|  |  |  |  |  | Initial DSCG (60)  Final DSCG (42) | *1) Increment* *in height over 18 months:* 8.80 cm; 95% CI: +8.35 to +9.25 | n = 16 (27%) | NR |
| Verberne 1997(33) | Adequate | Adequate | Double-blind | Height measured using stadiometer. | Salm 50 mcg bd (32) | ***GV:***6.1; 95% CI: +5.3 to +6.9 | 7 | None |
|  |  |  |  |  | BDP 200 µg bd  (35) | ***GV:***4.7; 95% CI: +4.0 to +5.3 | 3 | None |

BDP: beclometasone diproprionate; BUD: budesonide; DSCG: sodium cromoglicate; DPI: dry powder inhaler; FP: fluticasone propionate; MDI: Metered dose inhaler; MF: mometasone furoate, Salm: Salmeterol
