# Supplementary material for: Impact of Inhaled Corticosteroids on Growth in Children with Asthma: Systematic Review and Meta-Analysis
Source: PLoS One. 2015 Jul 20;10(7):e0133428. doi: 10.1371/journal.pone.0133428 (PMC4507851; doi:10.1371/journal.pone.0133428)
Supplement: S4 Table — (DOCX) [file pone.0133428.s007.docx]

**S4 Table: Study Validity and Growth Outcomes in Observational Studies**

| **Study** | **Outcome; ascertainment and validation of adverse effect** | **Definition of ICS use; verification of actual exposure; follow up period or study duration** | **Statistical adjustments for confounding factors** | **Results** |
| --- | --- | --- | --- | --- |
| **Agertoft & Pedersen 2000** **(34)** | Outcome – adult height (mean of three measurements with a Harpenden stadiometer), | Outpatient users of budesonide in longitudinal study, mean daily dose 412 mcg  Follow up mean of 9.2 years of treatment to adult height | None | Final adult height:  Budesonide group: 173.2 cm (SD 9.5)  Asthma, non ICS users: 173.9 cm (SD 10.1) |
| **Merkus 1993** **(35)** | Regular measurements by one investigator. | Outpatient records, with compliance check. ICS users had salbutamol 0.2mg + budesonide 0.2mg 3 times a day | Matched for sex, age, height. | Mean difference in growth velocity in 2.5 year follow-up, ICS vs. controls: -0.44 (95% CI -1.25 – 0.37) |
| **Silverstein 1997** **(36)** | Standing height, shoes removed. Same study nurse, single calibrated stadiometer. Measurements repeated five times, and mean analysed. | Nurse review of all outpatient and inpatient medical records from asthma onset to adult height. | Adjusted for survey-reported mid-parental height. | Mean difference measured adult height between ICS users and non-users:  -0.9 cm; 95% CI: -3.8 - 2.0 cm |
